# Retained Lead Fragments in Superior Vena Cava and Early Post-Transplant Outcomes: A Single Center Preliminary Retrospective Study

**DOI:** 10.3390/biomedicines13102509

**Published:** 2025-10-15

**Authors:** Federica Mazzone, Lorenzo Giovannico, Vincenzo Ezio Santobuono, Giuseppe Fischetti, Domenico Parigino, Luca Savino, Claudia Leo, Giuseppe Cristiano, Aldo Domenico Milano, Andrea Igoren Guaricci, Massimo Padalino, Marco Matteo Ciccone, Tomaso Bottio

**Affiliations:** 1 Cardiac Surgery Unit, Department of Precision and Regenerative Medicine and Ionian Area (DiMePRe-J), University of Bari Medical School, Piazza Giulio Cesare 11, 70124 Bari, Italy; 2 University Cardiology Unit, Interdisciplinary Department of Medicine, University of Bari Aldo Moro, 70121 Bari, Italyandrea.guaricci@gmail.com (A.I.G.);

**Keywords:** heart transplantation, cardiac implantable electronic devices, ICD, pacemaker, lead retention, early mortality, superior vena cava, postoperative outcomes, survival analysis, cardiovascular surgery

## Abstract

**Background/Objectives**: Retained fragments of cardiovascular implantable electronic device (CIED) leads are frequently observed after orthotopic heart transplantation (OHT), but their clinical relevance remains unclear. **Methods**: We conducted a single-center, retrospective study of 179 adult patients who underwent OHT between January 2022 and January 2025. Post-transplant imaging was used to identify retained lead fragments. Patients were grouped based on the presence or absence of retained leads. The primary endpoint was all-cause mortality at 30, 90, and 150 days post-transplant. Survival analysis was performed using Kaplan–Meier estimates and Cox proportional hazards modeling. **Results**: Among 112 patients with pre-transplant CIEDs, 18 (16%) had retained intravascular lead fragments. These patients had significantly lower survival at 30 days (66.7% vs. 94.7%), 90 days (61.1% vs. 90.3%), and 150 days (55.0% vs. 83.5%) compared to those without retained fragments (log-rank *p* = 0.002). The presence of retained leads was independently associated with increased mortality (HR: 3.71; 95% CI: 1.55–8.84; *p* = 0.003), even after adjusting for potential confounders. **Conclusions**: Retained CIED lead fragments are independently associated with higher early post-transplant mortality. These findings support the need for individualized intraoperative strategies to mitigate hardware-related risks in high-risk transplant candidates.

## 1. Introduction

Heart transplant recipients frequently have a history of cardiovascular implantable electronic devices (CIEDs), including implantable cardioverter-defibrillators (ICDs), cardiac resynchronization therapy (CRT) systems, and pacemakers. These devices are integral to the therapeutic management of advanced heart failure, particularly in patients at elevated risk of sudden cardiac death or with electromechanical dyssynchrony. Their clinical efficacy often depends on the implantation of multiple transvenous leads, each dedicated to distinct functions such as atrial sensing, ventricular pacing, defibrillation, or biventricular resynchronization [1]. During orthotopic heart transplantation (OHT), complete extraction of chronically implanted leads can be challenging; standard surgical practice often involves cutting the transvenous leads at the level of the superior vena cava (SVC) and leaving remnants in situ. As a result, retained pacemaker/ICD lead fragments within the central venous circulation are common, reported in up to ~30% of transplant recipients [2]. Retained intravascular leads represent a persistent foreign body and potential source of complications. Chronically indwelling leads can promote endothelial trauma, venous intimal hyperplasia, and fibrosis, ultimately predisposing to stenosis or thrombosis of the central venous system, that may culminate in clinically significant superior vena cava (SVC) obstruction. While large-scale studies suggest that severe venous stenosis remains relatively uncommon in patients with chronic ICD leads [3], the increased use of transvenous devices, dual-coil systems, and multiple lead implantations has amplified the risk of benign SVC syndrome in selected populations [4]. This risk becomes particularly relevant in the heart transplant setting, where the SVC is manipulated surgically and anastomosed, potentially exacerbating pre-existing venous abnormalities. In addition to thromboembolic complications, retained lead fragments pose other clinical risks. Such cases highlight the potential for retained device materials to cause serious infectious complications long after transplant higher [5]. Furthermore, residual lead materials may interfere with routine post-transplant care. For instance, the presence of metallic lead fragments often precludes magnetic resonance imaging (MRI) due to safety concerns, limiting advanced imaging options in these patients [6]. Retained leads within the SVC or great veins might also complicate repeated endomyocardial biopsy or central venous access, as they can alter venous anatomy and increase procedure complexity [7]. These considerations underscore that even “abandoned” leads are not entirely inert and may contribute to morbidity.

In this context, we sought to evaluate the impact of retained pacemaker/defibrillator lead fragments on short-term outcomes after heart transplantation. Our aim is to clarify whether abandoned device leads confer additional risk in the critical post-transplant phase, thereby guiding future management strategies for patients with cardiac devices undergoing transplantation.

## 2. Subjects and Methods

▪ Study Design and Population

**Figure 1:**
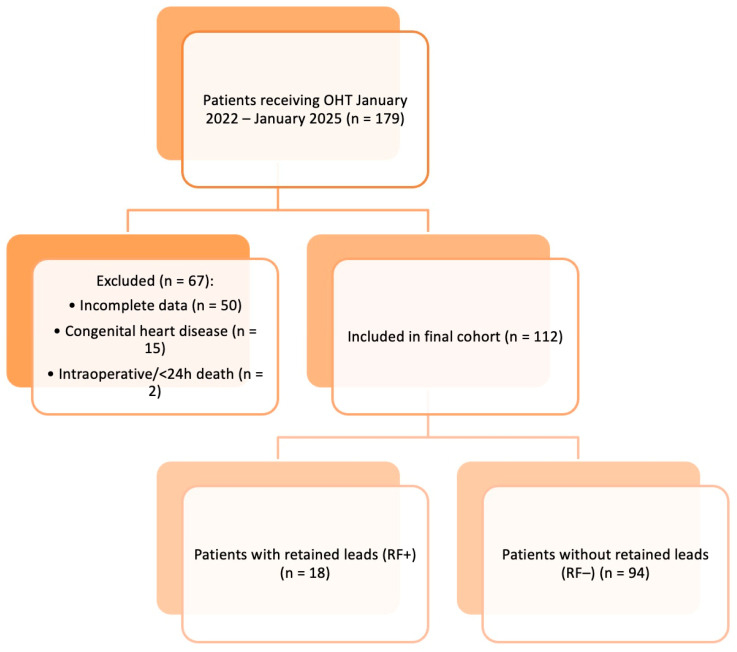
Flowchart of patient selection for orthotopic heart transplantation (OHT) between January 2022 and January 2025. Of 179 initial patients, 67 were excluded due to intraoperative death, death within 24 h, incomplete data, or congenital heart disease. The final cohort included 112 patients, subdivided into 18 with retained intracardiac leads (RF+) and 94 without retained leads (RF−).

This retrospective cohort observational study included all adult patients (aged ≥18 years) who underwent orthotopic heart transplantation (OHT) at Cardiac Surgery Unit (Figure 1), Department of Precision and Regenerative Medicine and Ionian Area (DiMePRe-J) of University of Bari between January 2022 and January 2025. Patient data were retrieved from the institutional transplant registry and electronic medical records.

▪ Inclusion and Exclusion Criteria
  Patients were eligible for inclusion if they:
    - Underwent OHT during the study period;
    - Had retained CIED lead fragments within the superior vena cava (SVC), as confirmed by post-transplant imaging (chest radiography or computed tomography);
    - Had complete clinical data and at least 150 days of post-transplant follow-up.
  Patients were excluded if they:
    - Died intraoperatively or within 24 h of transplantation (n = 2);
    - Had incomplete clinical documentation (n = 50);
    - Had congenital heart disease requiring complex vascular reconstruction (n = 15).

▪ Data Collection

Data collected included demographic information, comorbidities, type and duration of pre-transplant CIED implantation, number and type of leads, operative details, and post-transplant outcomes. Retained lead fragments were defined as visible remnants of transvenous pacemaker or defibrillator leads left in situ during surgery.

▪ Outcomes

The primary endpoint was all-cause mortality at 30, 90, and 150 days post-transplant. Survival outcomes were compared between patients with and without retained lead fragments. Secondary endpoints included demographic and clinical characteristics associated with lead retention.

▪ Immunosuppression Therapy

Our institutional immunosuppression protocol consists of induction and maintenance phases. Induction therapy begins at the time of aortic unclamping with 1 g of intravenous methylprednisolone. At the end of surgery, rabbit anti-thymocyte globulin (rATG) is initiated at a dose of 25 mg/day (diluted in 50 mL saline and infused at 2.1 mL/h) after premedication with 1000 mg paracetamol and 10 mg chlorphenamine. On postoperative days (POD) 1–2, methylprednisolone is continued at 125 mg IV three times daily. On POD 3, a calcineurin inhibitor (tacrolimus) is introduced at the minimal dose (0.5 mg twice daily). rATG is discontinued on POD 5. Maintenance therapy includes tacrolimus with target trough levels of 8–10 ng/mL, mycophenolate mofetil (500–1000 mg twice daily) or alternatively an mTOR inhibitor (everolimus, target trough 4–6 ng/mL), and prednisone (initially 25 mg/day with progressive taper, discontinued by 12 months post-transplant in the absence of rejection episodes).

▪ Lead Removal Technique

Lead removal was systematically performed surgically at the end of the orthotopic heart transplantation procedure, after completion of the anastomoses and hemostasis. The transvenous leads were transected at the time of cardiectomy, usually at the level of the superior vena cava–right atrial junction, to allow safe explantation of the recipient heart.

When feasible, gentle manual traction was applied under direct vision to extract as much of the lead as possible through the venous entry site. No percutaneous or powered extraction tools were employed. Residual fragments were intentionally left in situ when significant fibrotic adhesions, venous wall incorporation, or excessive resistance were encountered, as further traction was considered to carry a high risk of vascular laceration, hemorrhage, or superior vena cava injury.

The decision to terminate extraction attempts was made by the operating surgeon in order to minimize operative time and avoid hemodynamic instability. The length and location of retained fragments were documented intraoperatively for postoperative monitoring and follow-up.

## 3. Statistical Analysis

Statistical analyses were performed using R software (version 4.3.0). Continuous variables are presented as median and interquartile range, while categorical variables are reported as frequencies and percentages. Comparisons of categorical variables were performed using Chi-square or Fisher’s exact test as appropriate, and continuous variables were compared using Student’s *t*-test or Mann–Whitney U test based on data distribution. Variables with *p* < 0.10 in univariate analysis were entered into the multivariate Cox proportional hazards model. All statistical tests were considered significant at a *p*-value < 0.05. Survival was estimated using the Kaplan–Meier method and compared between groups with the log-rank test. Survival curves were graphically represented, including the risk table and the *p*-value from the log-rank test to assess statistical significance. To quantify the relative risk of mortality associated with the presence of retained fragments, a Cox proportional hazards regression model was used. The hazard ratio (HR) and its 95% confidence interval were calculated for the group with fragments compared to the group without.

## 4. Results

Out of a total of 179 heart transplant recipients meeting inclusion criteria, 112 patients (62.5%) had a history of CIED implantation prior to transplantation. Among them, 18 patients (16%) were found to have retained intravascular lead fragments in the post-transplant period.

### 4.1. Risk Factor Analysis

No preoperative demographic or clinical factors were significantly associated with the presence of retained lead fragments. Detailed patient characteristics and statistical comparisons are reported in Table 1.

### 4.2. Outcome Analysis

The results showed that 150-day survival was significantly lower in patients with retained electrode fragments compared to those without. Specifically, the log-rank test demonstrated a significant difference between the groups (*p* = 0.002). Thirty-day survival was 94.7% in RF− patients compared to 66.7% in RF+ patients (Table 2). On univariate analysis, none of the preoperative clinical factors showed a statistically significant association with the presence of retained fragments or early mortality.

To quantify the relative risk of mortality associated with the presence of retained fragments, a Cox proportional hazards regression model was used. The hazard ratio (HR) and its 95% confidence interval were calculated for the group with fragments compared to the group without. The Cox model results further quantify the increased risk associated with retained fragments, supporting the conclusion that their presence is a negative prognostic factor after transplantation. Early postoperative mortality was of particular interest given prior reports suggesting that the most severe complications associated with retained CIED lead fragments tend to occur within the first weeks following transplantation.

Cox Proportional Hazards Model

- ***Hazard Ratio (HR):** 3.71*
- ***95% Confidence Interval:** 1.55–8.84*
- ***p-value:** 0.00316*

The accompanying table details the exact survival probabilities, confidence intervals, number at risk, and number of events at each time point. Multivariate analysis using the Cox model confirmed that the presence of retained electrode fragments was associated with a significantly increased risk of mortality within the early post-transplant period.

## 5. Discussion

**Figure 2:**
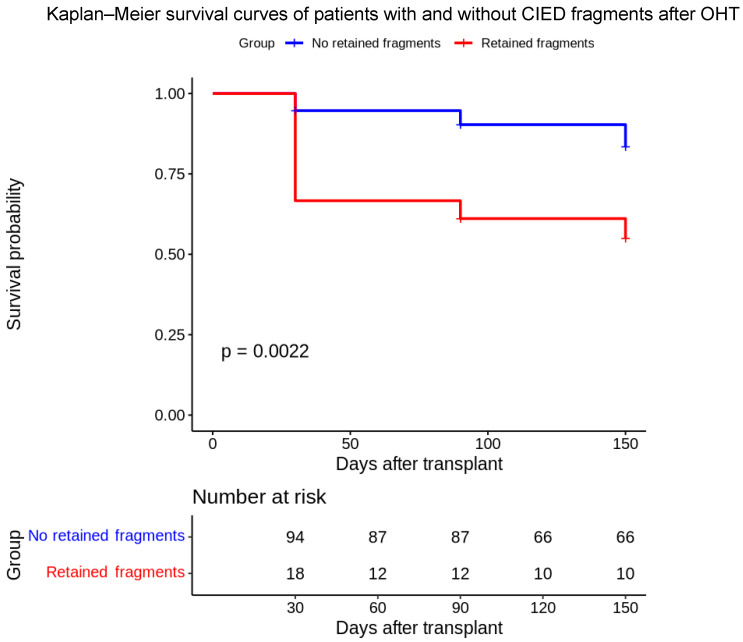
The Kaplan–Meier plot above visually demonstrates the difference in survival between the two groups, with the blue curve representing patients without retained fragments (higher survival) and the red curve representing those with retained fragments (lower survival).

This retrospective, single-center study provides new evidence that the presence of retained cardiovascular implantable electronic device (CIED) lead fragments following orthotopic heart transplantation (OHT) is independently associated with increased short-term mortality. Patients with retained intravascular leads exhibited significantly lower survival at 30, 90, and 150 days post-transplantation. Survival at 30, 90, and 150 days was significantly lower in patients with retained fragments compared to those without (Figure 2) (30 days: 66.7% vs. 94.7%; 90 days: 61.1% vs. 90.3%; 150 days: 55.0% vs. 83.5%; log-rank *p* = 0.002). The presence of retained fragments was associated with an almost four-fold increased risk of mortality (HR 3.71; 95% CI: 1.55–8.84; *p* = 0.003). The association remained robust after multivariate adjustment, with a hazard ratio of 3.71 (95% CI: 1.55–8.84; *p* = 0.003), highlighting retained leads as a negative prognostic factor during the critical early postoperative period.

These findings stand in contrast to several earlier studies that reported high rates of lead retention post-transplant but did not observe a corresponding increase in mortality. Martin et al. [2] retrospectively analyzed 138 heart transplant recipients and found a 27% prevalence of retained device fragments. Their study included a survival analysis, which showed no significant difference in long-term survival between patients with and without retained leads (log-rank *p* = 0.60) [2]. Similarly, Pettit et al. [8] examined 85 heart transplant recipients and reported that 30% had retained pacemaker or ICD components. They also found no statistically significant difference in survival (log-rank *p* = 0.44) over a median follow-up of 3.6 years [8]. Austin et al. [9] further reinforced this interpretation in a 2017 single-center study involving 114 heart transplant recipients, 40 of whom (35.1%) had retained CIED lead fragments. No major MRI-related complications or deaths were reported, and Kaplan–Meier analysis showed no significant difference in survival between patients with and without retained leads during a median follow-up of 4.2 years. On this basis, the authors concluded that retained lead fragments are not associated with increased morbidity or mortality, further supporting the notion that these remnants are clinically inert [9]. Our findings contrast with several previous studies that, while documenting a high prevalence of retained CIED components after heart transplantation, did not report significant differences in long-term survival between patients with and without retained fragments. In particular, Martin et al. (27% retention; log-rank *p* = 0.60), Pettit et al. (30%; log-rank *p* = 0.44), and Austin et al. (35.1%; median follow-up 4.2 years) [2,8,9] found no impact on mortality or morbidity during extended follow-up. In contrast, our study was specifically designed to explore early postoperative outcomes and demonstrates significantly lower survival at 30, 90, and 150 days in patients with retained fragments, with an independent association with increased mortality risk (HR 3.71). The novelty of our work lies precisely in its focus on the immediate post-transplant window, where most deaths in the RF+ group occurred—a temporally distinct dynamic likely driven by thrombotic, inflammatory, and mechanical mechanisms that become clinically relevant in the early postoperative phase.

Based on these findings, groups concluded that retained leads did not adversely impact patient outcomes. However, a key distinction lies in the timing of events captured. The above studies primarily evaluated long-term survival, whereas our analysis specifically focused on early postoperative mortality. In our cohort, most deaths among patients with retained leads occurred within the first 30 days, a time window not specifically highlighted in prior studies. This distinction is clinically relevant: while retained leads may remain asymptomatic in stable patients over time, their presence in the immediate post-transplant setting—particularly in critically ill patients—may contribute to adverse outcomes through thrombotic, inflammatory, or mechanical mechanisms. It should be noted that this excess mortality could not be attributed to any obvious baseline differences. In this series, patients with retained leads were comparable to others in terms of age, body size, number and type of leads, and implant duration, as well as preoperative diagnosis. This finding suggests that the mere presence or existence of hardware, or its retention in a clinical context, is associated with elevated risk.

Several mechanisms could explain why retained leads are associated with early post-transplant mortality. In practice, lead extraction is often deferred in the sickest patients—those undergoing urgent, complex, or prolonged transplant procedures (especially if on mechanical circulatory support or with extensive lead fibrosis). Because heart transplantation is often performed emergently, there may be no opportunity to deploy specialized lead-extraction tools or teams, making complete device removal unfeasible. While avoiding a difficult extraction may be clinically justified to shorten surgical time and avoid hemodynamic instability, the trade-off is that foreign material is left in the vasculature during a critical period. This retained hardware can act as a nidus for thrombus and inflammation [10]. For instance, Holzhauser et al. found that heart transplant patients with retained lead fragments had more than double the incidence of upper-extremity deep vein thrombosis within one year (42% vs. 21% in those without fragments) [7]. Beyond thrombosis, retained leads in an immunosuppressed host may serve as foci for infection [5].

Heart transplantation remains the gold standard treatment for selected patients with end-stage heart failure, as emphasized in the most recent consensus statements from the International Society for Heart and Lung Transplantation (ISHLT), the European Society of Cardiology (ESC) Heart Failure Association (HFA), and the European Association for Cardio-Thoracic Surgery (EACTS) [11,12,13]. Advances in surgical technique and perioperative management have improved survival, yet early post-transplant mortality remains a major concern, particularly in high-risk recipients [14]. In this context, hardware-related complications such as retained cardiovascular implantable electronic device (CIED) fragments have received comparatively little attention.

From a management standpoint, these findings question the prevailing assumption that retained leads are benign. Current HRS and ESC/HFA consensus statements recognize the complexity of lead extraction and recommend individualized decision making [15,16,17]. The ELECTRa registry has demonstrated that transvenous extraction, when performed in high-volume centers, is feasible and safe [18]. In selected transplant candidates—particularly those undergoing elective procedures—consideration of pre-transplant extraction or advanced intraoperative removal strategies may therefore be warranted. Moreover, tailored post-transplant surveillance (systematic imaging for thrombosis, careful infection monitoring, and individualized anticoagulation strategies) could help mitigate risks in patients with retained hardware.

## 6. Conclusions

In conclusion, retained CIED lead fragments following heart transplantation are associated with a significantly increased risk of early postoperative mortality. While prior studies have generally viewed these fragments as clinically inert, our findings suggest otherwise—particularly in patients undergoing complex or high-risk procedures where lead extraction is deferred. These results underscore the need for a more individualized approach to intraoperative lead management and support further investigation into strategies for safe extraction or mitigation of retained hardware complications in transplant recipients.

### Study Limitations

This study is limited by its retrospective, single-center design, which may introduce selection and information bias and restrict the generalizability of the findings to other institutions with different surgical expertise or patient populations. The number of patients with retained leads was relatively small (n = 18), which reduces the statistical power and increases the risk of type II error, particularly for less frequent outcomes. Although multivariate analysis was performed, unmeasured confounders—including intraoperative hemodynamics, degree of venous fibrosis, and donor–recipient procedural complexity—may have influenced outcomes. Furthermore, systematic post-transplant imaging to assess venous obstruction or thrombus burden was not routinely performed, and biological parameters such as inflammatory markers were not consistently collected, which may have provided further mechanistic insight into the observed association between lead retention and early mortality. Future multicenter prospective studies with standardized data collection are needed to confirm and expand these findings.

Nevertheless, the statistical strength of the association between retained leads and early mortality, the rigor of the survival analysis, and the internal consistency of the dataset support the validity of our conclusions.

## Figures and Tables

**Table 1:** None of the analyzed preoperative clinical factors were significantly associated with the presence of retained catheter fragments and preoperative diagnosis (all *p* > 0.2). The type of underlying heart disease was not a significant predictor of lead retention at the time of transplantation. *p* value for Chi^2^ or Fisher’s Exact, as appropriate.

Patient Characteristics	Patients with RF (N = 18)	Patients Without RF (N = 94)	*p*-Value
**Age (mean ± SD)**	61.89 ± 5.74 years	59.78 ± 10.29 years	0.2197
**BSA (mean ± SD)**	1.82 ± 0.16 m^2^	1.85 ± 0.19 m^2^	0.5961
**Female Sex (%)**	31.6%	12.9%	0.0935
**Hypertension (%)**	66.0%	66.7%	0.954
**Diabetes (%)**	28.7%	33.3%	0.695
**Peripheral Vascular Disease (PVD) (%)**	22.3%	22.2%	0.991
**COPD (%)**	22.3%	33.3%	0.322
**Atrial Fibrillation (%)**	28.7%	22.2%	0.574
**≥2 Leads (%)**	52.6%	35.5%	0.2536
**Dual Coil ICD (%)**	47.4%	29.0%	0.3214
**ICD implant >18 months (%)**	68.4%	52.7%	0.3155
**Dilated cardiomyopathy**	44.4%	42.6%	1.0
**Hypertrophic cardiomyopathy**	5.6%	2.1%	0.4119
**Ischemic heart disease**	33.3%	24.5%	0.6221
**Valvular disease**	11.1%	4.3%	0.2467
**Myocarditis**	5.6%	0.0%	0.1607

**Table 2:** Group 1: Patients without retained fragments; Group 2: Patients with retained fragments.

Time (Days)	Group	Survival Probability	95% CI (Lower)	95% CI (Upper)	Number at Risk	Number of Events
30	1	94.7%	90.3%	99.3%	94	5
90	1	90.3%	84.5%	96.5%	87	4
150	1	83.5%	75.9%	91.9%	66	5
30	2	66.7%	48.1%	92.4%	18	6
90	2	61.1%	42.3%	88.3%	12	1
150	2	55.0%	36.1%	83.9%	10	1

## Data Availability

The data presented in this study are available upon request from the corresponding author.
